# Editorial: The Metabolic Challenges of Immune Cells in Health and Disease

**DOI:** 10.3389/fimmu.2015.00293

**Published:** 2015-06-04

**Authors:** Christian Frezza, Claudio Mauro

**Affiliations:** ^1^MRC Cancer Unit, Hutchison/MRC Research Centre, University of Cambridge, Cambridge, UK; ^2^William Harvey Research Institute, Barts and The London School of Medicine and Dentistry, Queen Mary University of London, London, UK

**Keywords:** immunometabolism, metabolic disease, T cells, B cells, macrophages

Only few years ago, scientists had to struggle to convince audiences and editors that cell metabolism and biochemistry were not boring, let alone persuade the scientific community that alterations of the metabolic machinery could underpin human diseases (1). We hear from more senior scientists (we were only undergraduate students at the time, more often than not dreading our studies of glycolysis, Krebs cycle, electron transport chain, etc., on the “famous” Lehninger text-book) that publishing the first paper on c-Myc-mediated transcriptional control of the metabolic enzyme lactate dehydrogenase as a key mechanism for cancer transformation (2) or lymphocyte survival and activation via TCR-dependent regulation of nutrient uptake and utilization (3, 4) was not easy at all. Indeed, they had to overcome the preconception of a well-established scientific community that, for the last few decades, had believed in the supremacy of molecular biology and genetics as experimental tools for understanding cellular mechanisms and disease processes.

In 2015, metabolism is the heart of an ever growing body of studies, spanning the fields of cancer, stem cells, and, as highlighted in this series of review articles, immunology and metabolic diseases. This unexpected renaissance in the field of metabolism stands on the shoulders of giants. Indeed, scientists of the caliber of Warburg, Krebs, and Mitchell, just to name a few, spent their entire lives exploring the intricacies of cell metabolism. Not only had they elucidated the pathways for utilization of glucose and other nutrients for the generation of ATP but had also initiated the modern and fashionable concept of integration of metabolic processes with diseases and immune-regulation. As described by Nagy and Haschemi (5), Kempner and Peschel proposed the idea of a tight link between metabolism and inflammation, the modern so-called immunometabolism, as far back as 1930s. Unfortunately, the whole field of metabolism was relegated to the margins of modern research for long time, being considered irrelevant for addressing more important questions, such as how proliferation, differentiation, and cell death, are regulated in the cell. This obnubilation lasted until the realization that all these processes have distinct metabolic requirements and that impairing metabolism could perturb them. We now know that signaling pathways directly control specific metabolic pathways and enzymes, and vice versa, and even more astonishingly, that intermediates of metabolism, such as lactate or succinate, or metabolic enzymes (i.e., GAPDH or PFKFB3) can regulate gene expression, protein translation, or indeed entire processes, such as endothelial sprouting.

The studies on immunometabolism that we present here encompass both cellular and systemic aspects of disease. At a cellular level, immunometabolism studies show how intracellular metabolic pathways activated downstream of growth factors and cytokines control immune cell functions. On an organismal level, immunometabolism investigates how immune cells regulate the homeostasis of metabolic tissues and how they contribute to the process of metabolic diseases, including obesity and type II diabetes. This collection contains 10 review articles that cover important and emerging aspects in both of these branches of immunometabolism.

At the cellular level, Howie et al. (6) focus on the mechanisms of nutrient sensing in T cells and how these integrate with TCR and cytokine signals via the mTOR pathway to determine distinct differentiation pathways toward effector or regulatory T cell (Treg) subsets. Going deeper into the biology of Treg lymphocytes, Coe et al. (7) describe recent findings on the unique metabolic needs of Treg as compared to effector T cells (Teff), with a particular focus on mTOR-mediated control of metabolism in these T cell subsets. Schurich and Henson (8) discuss the emerging view that as a consequence of viral infection and antigenic load, CD8^+^ T cells can become senescent or exhausted. These are distinct fates of a T cell, sustained by different metabolic programs, which in turn dictate opposing outcomes during immune responses. Nagy and Haschemi (5) illustrate the metabolic changes that take place in macrophages upon LPS-induced activation and polarization. They then focus on how the pentose phosphate pathway is regulated during LPS- versus IL-4-induced polarization of macrophages and may be of importance in the provision of both nucleotide precursors and redox-equivalents determining different cell fates and types of immune response. Finally, Jones and Bianchi (9) give an overview of some recent key examples of metabolic control of biological processes beyond cellular proliferation. In particular, the roles of intermediates of metabolism in the control of gene expression, of metabolic enzymes in the regulation of protein translation and cellular differentiation, and of aerobic glycolysis in epigenetic determination of trained immunity are discussed.

At a more systemic level, Wang et al. (10) offer their views on the possible interplay between tumor cells and immune cells in the tumor microenvironment. Tumor cells may compete for nutrients with some immune cells, thereby compromising their function. Tumor cells may also become metabolically symbiotic with other immune cells, which in turn may provide signals for tumor growth. Gerriets and MacIver (11) overview the well-established link between nutritional status and immune cell function. They pay particular attention to the signals linking nutrient stress to T cell metabolic adaptation and how this crosstalk may result in low-grade inflammation leading to metabolic syndrome as a consequence of obesity or increased risk of mortality by infectious diseases as a consequence of malnutrition. The connection between immune cells and metabolic diseases is further discussed by Capasso et al. (12), who ask questions about the possible crosstalk of B cells with the adipose tissue in homeostatic conditions and during obesity; however, this field is currently understudied, leaving us with more questions than answers. Continuing on the same issue, Mauro et al. (13) discuss the clinical evidence of the association that exists between increased incidence of obesity worldwide and increased prevalence and severity of cognitive disorders. They speculate that systemic metabolic imbalance can have direct consequences on the integrity and function of the blood–brain barrier, thereby leading to the insurgence of cerebrovascular and neurodegenerative pathologies; however, the mechanistic links are unknown at present. Finally, Tannahill et al. (14) discuss the importance of macrophages in the pathogenesis of multiple sclerosis, the metabolic changes behind macrophage polarization, and how macrophage metabolic re-education could be used in the future for the treatment of multiple sclerosis.

In conclusion, these are exciting times for the discovery of the many mechanisms of integration between metabolic and signaling pathways as ways to determine cell fates and types of immune response. These are also exciting times for those who are investigating the metabolic crosstalk between immune cells and stromal cells during homeostasis and in diseased tissues. We are hopeful that gaining deeper understanding of how metabolism and signaling pathways coordinate with each other will lead to new perspectives on disease mechanisms and, ultimately, to the development of novel therapeutic tools.

## Conflict of Interest Statement

The authors declare that the research was conducted in the absence of any commercial or financial relationships that could be construed as a potential conflict of interest.
